# Glucagon-Like Peptide-1 Receptor Agonists Reduce Liver Stiffness in a Pediatric Metabolic Dysfunction-Associated Liver Disease Cohort and Normalize Disease-Associated Core Gene Expression in an Experimental Model

**DOI:** 10.7759/cureus.106280

**Published:** 2026-04-01

**Authors:** Ella Findling, Terrence Bissoondial, Prakash Narayan

**Affiliations:** 1 Science, George W. Hewlett High School, Hewlett, USA; 2 Bioinformatics, Nodes and Edges LLC, Raleigh, USA

**Keywords:** glp-1 receptor agonists, hepatic fibrosis, hepatic inflammation, hepatic stellate cells, liver steatosis, masld, pediatric gastroenterology, pediatric liver disease, transcriptomic profiling, transient elastography (fibroscan)

## Abstract

Metabolically-dysfunction-associated steatotic liver disease (MASLD) is one of the most common chronic liver diseases in children and is strongly associated with obesity and insulin resistance. In this study, we evaluated the clinical effects of glucagon-like peptide-1 (GLP-1) receptor agonist (RA) therapy in a small, de-identified cohort of pediatric patients with MASLD and investigated potential molecular mechanisms using publicly available transcriptomic datasets from models of liver disease. Longitudinal FibroScan® (Echosens, Paris, France) measurements from seven pediatric patients treated with GLP-1 RAs demonstrated significant reductions in controlled attenuation parameter scores, transient elastography scores, and aspartate aminotransferase levels, indicating improvements in liver steatosis, liver stiffness, and liver inflammatory profiles, respectively. To explore potential mechanisms underlying these observations, we analyzed transcriptomic datasets from methionine-choline-deficient and high-fat diet (HFD) murine models of liver disease. A pattern-matching algorithm identified a core set of 10 genes consistently upregulated in both models and downregulated with GLP-1 RA treatment in the HFD model. These genes are enriched in extracellular matrix remodeling, inflammatory signaling, and fibrogenic pathways associated with hepatic stellate cell activation. Collectively, these findings engender the hypothesis that GLP-1 RAs may prove therapeutic in pediatric MASLD by attenuating inflammation and fibrosis. Although limited by a small cohort size, this integrated clinical-transcriptomic approach supports further investigation of GLP-1 RAs as a fast-follower therapeutic strategy for pediatric MASLD.

## Introduction

Metabolically-dysfunction-associated steatotic liver disease (MASLD) is the most common pediatric chronic liver disease, affecting 5-11% of all children, with rates rising to 30-50% in children with obesity [1]. Left untreated, MASLD can progress to metabolic dysfunction-associated steatotohepatitis (MASH) and fibrosis, and increase the risk for early hepatocellular carcinoma, especially in children with obesity and insulin resistance [2-4]. In addition to attendant symptoms and comorbidities that effectively steal childhood, pediatric MASLD is associated with a 40-fold increased risk of early mortality and need for a liver transplant in young adults [5].

The MASLD continuum is driven primarily by insulin resistance, which promotes the accumulation of hepatic fat [6,7]. With time, metabolic stress and steatosis trigger inflammatory pathways and cellular injury, promoting the transition to MASH or steatosis with inflammation [8,9]. Chronic injury activates hepatic stellate cells (HSCs), which start secreting excessive matrix-disrupting structures within the liver, impairing blood flow and stiffening liver tissue [10-13].

Management of pediatric MASLD relies on lifestyle and diet modifications as frontline therapy [14]. Nevertheless, not only is sustaining such changes challenging, but many patients are refractory to such changes. Over the last decade, adult patients with metabolic diseases have benefited from novel therapeutics, including glucagon-like peptide-1 (GLP-1) receptor agonists (RAs), which have positively impacted end organs, including the kidneys and liver [15,16].

Our overarching hypothesis is that GLP-1 RAs are beneficial in pediatric MASLD via downregulation of pathways driving the MASLD-MASH-fibrosis continuum. In the present study, we evaluated the use of GLP-1 RAs in a small, de-identified pediatric MASLD cohort and investigated its putative mechanism of action by analyzing publicly available transcriptomic datasets from murine models of liver disease.

## Materials and methods

Clinical data

To evaluate the efficacy of GLP-1 RA therapy, we analyzed longitudinal FibroScan® (Echosens, Paris, France) data from a cohort of seven pediatric patients diagnosed with metabolic dysfunction-associated steatotic liver disease (MASLD). De-identified clinical data were obtained from Northwell Health, New York, United States, following approval by the Northwell Health Institutional Review Board (approval number: 25-1020). All data were collected retrospectively from electronic medical records and de-identified prior to analysis.

Inclusion criteria were age less than 18 years at treatment initiation, class 2 or higher obesity, clinical diagnosis of MASLD, and a minimum of three months of treatment with a GLP-1 RA. Patients were included only if both baseline (pre-treatment) and follow-up FibroScan measurements were available. Concomitant medications and comorbid conditions were recorded when available.

Noninvasive vibration-controlled transient elastography was used to assess hepatic steatosis and liver stiffness [17]. Hepatic steatosis was estimated using the controlled attenuation parameter (CAP), expressed in dB/m, and liver stiffness was measured using transient elastography (TE), expressed in kPa. FibroScan measurements were obtained as part of routine clinical care, and reported values represent the median of multiple valid measurements obtained during each examination according to standard clinical protocols.

Liver function tests, including aspartate aminotransferase (AST) and alanine aminotransferase (ALT), were obtained from routine blood draws performed as part of clinical management. The AST/ALT ratio (de Ritis ratio) [18] was calculated for each patient at baseline and follow-up time points. Body mass index (BMI), weight, and HbA1c values were also recorded when available to assess metabolic changes during therapy.

Missing data were not imputed; analyses were performed using available data only, and paired comparisons were conducted using patients with both baseline and follow-up measurements. Because of the small sample size, each patient served as their own control in longitudinal comparisons.

Transcriptomic analyses from MASLD models

Transcriptomic datasets were retrieved from the National Center for Biotechnology Information (NCBI) Gene Expression Omnibus [19]. Two specific datasets were selected: GSE267124, consisting of 12 mice fed a methionine-choline-deficient (MCD) diet to induce advanced fibrosis, and GSE243681, consisting of mice with high-fat diet (HFD)-induced obesity treated with a GLP-1 RA [20]. Raw mRNA counts were processed using iDEP 2.0 (South Dakota State University, Brookings, South Dakota, United States) for normalization and principal component analysis (PCA). A pattern-matching algorithm was used to identify core genes common to both studies. Core genes were fed to GepLiver [21] for evaluation of their expression across murine models of MASLD. Humanbase [22] and Gene Ontology (GO) Biological Process (BP) [23] were used to visualize core gene interaction and pathway activation signatures, respectively.

Data analysis

Calculations were made using Microsoft Excel version 2602 (Microsoft Corporation, Redmond, Washington, United States). Effects of GLP-1 RAs on clinical parameters were compared in GraphPad Prism version 11.0.0 (Dotmatics, Boston, Massachusetts, United States) using a paired two-tailed test or a Wilcoxon Signed-Rank test (non-parametric), and a Fisher’s exact test for responses. Differentially expressed genes from murine models were identified using a two-tailed t-test. A p<0.05 was considered statistically significant.

## Results

All patients, male and female, were less than 18 years of age at the onset of treatment, presented with class 2 or higher obesity, and were on GLP-1 RA (Wegovy or Ozempic) therapy for at least three months (Table 1). One patient was on Zepbound, which is a GLP-1 RA+glucose-dependent insulinotropic polypeptide (GIP). Comorbidities and concomitant medications are listed. Patients recorded a decrease in BMI and/or weight with therapy (Table 1). Prior to initiation of therapy, liver CAP scores were indicative of hepatic steatosis (upper limit of normal, 225 dB/m [24]). Liver TE scores, also elevated (upper limit of normal, 6.5 kPa [24]) prior to initiation of therapy, were reflective of liver stiffening; elevated liver function tests indicated inflammation (Table 1). 

**Table 1:** Patient level data for MASLD cohort prior to (salmon) and following (green) GLP-1 RA administration *Zepbound has GLP-1 RA+GIP activity. GLP-1: glucagon-like peptide-1; RA: receptor agonist; MASLD: metabolically-dysfunction-associated steatotic liver disease; AST: aspartate aminotransferase; ALT: alanine aminotransferase; CAP: controlled attenuation parameter; TE: transient elastography; GIP: glucose-dependent insulinotropic polypeptide

Variable	Patient 1	Patient 2	Patient 3	Patient 4	Patient 5	Patient 6	Patient 7
GLP-1 RA	Wegovy	Wegovy	Wegovy	Zepbound*	Wegovy	Wegovy	Ozempic
Concomitant Medications	Synthroid	Atenolol	-	Vitamin E	Vitamin E	-	Metformin
		Enalapril		Wellbutrin			
Age (years) at Onset	17	17	17	17	17	15	17
Sex	F	M	F	M	M	M	M
Comorbidities	Class 2 obesity	Class 2 obesity	Class 2 obesity	Class 2 obesity	Class 3 obesity	Class 2 obesity	Class 3 obesity
	Cong hypothyroid		Pre-diabetes	OSA	Dyslipidemia	Pre-diabetes	Diabetes
	Irregular menses		PCOS			Dyslipidemia	
pre-GLP1 RA	pre-GLP1 RA	pre-GLP1 RA	pre-GLP1 RA	pre-GLP1 RA	pre-GLP1 RA	pre-GLP1 RA	pre-GLP1 RA
BMI (kg/m2)	35.7	38.4	36.5	37.9	45.9	33.5	40.5
Weight (pounds)	198	223	201	254	283	240	298
AST (U/L)	90	24	31	91	59	147	54
ALT (U/L)	120	37	57	126	118	336	91
HbA1C	5.7	5.7	5.7	5.5	5.2	6.3	7.1
CAP (dB/m)	344	398	234	379	311	365	396
TE (kPa)	13	7.9	8.6	7.9	11.1	13.4	16.5
Months on GLP-1 RA	3	3	5	8	8	3	3
post-GLP-1 RA	post-GLP-1 RA	post-GLP-1 RA	post-GLP-1 RA	post-GLP-1 RA	post-GLP-1 RA	post-GLP-1 RA	post-GLP-1 RA
BMI (kg/m2)	33.6	36.5	33.1	36.2	40.4	31.4	39.5
Weight (pounds)	189	213	179	245	246	218	290
AST (U/L)	76	25	21	79	14	29	51
ALT (U/L)	122	44	20	98	12	75	59
HbA1C	5.4	5.4	5.1	5.2	5.1	5.3	6.6
CAP (dB/m)	240	339	190	305	260	308	295
TE (kPa)	10.8	6.9	6.6	7	4.5	10.2	11.1

**Figure 1:**
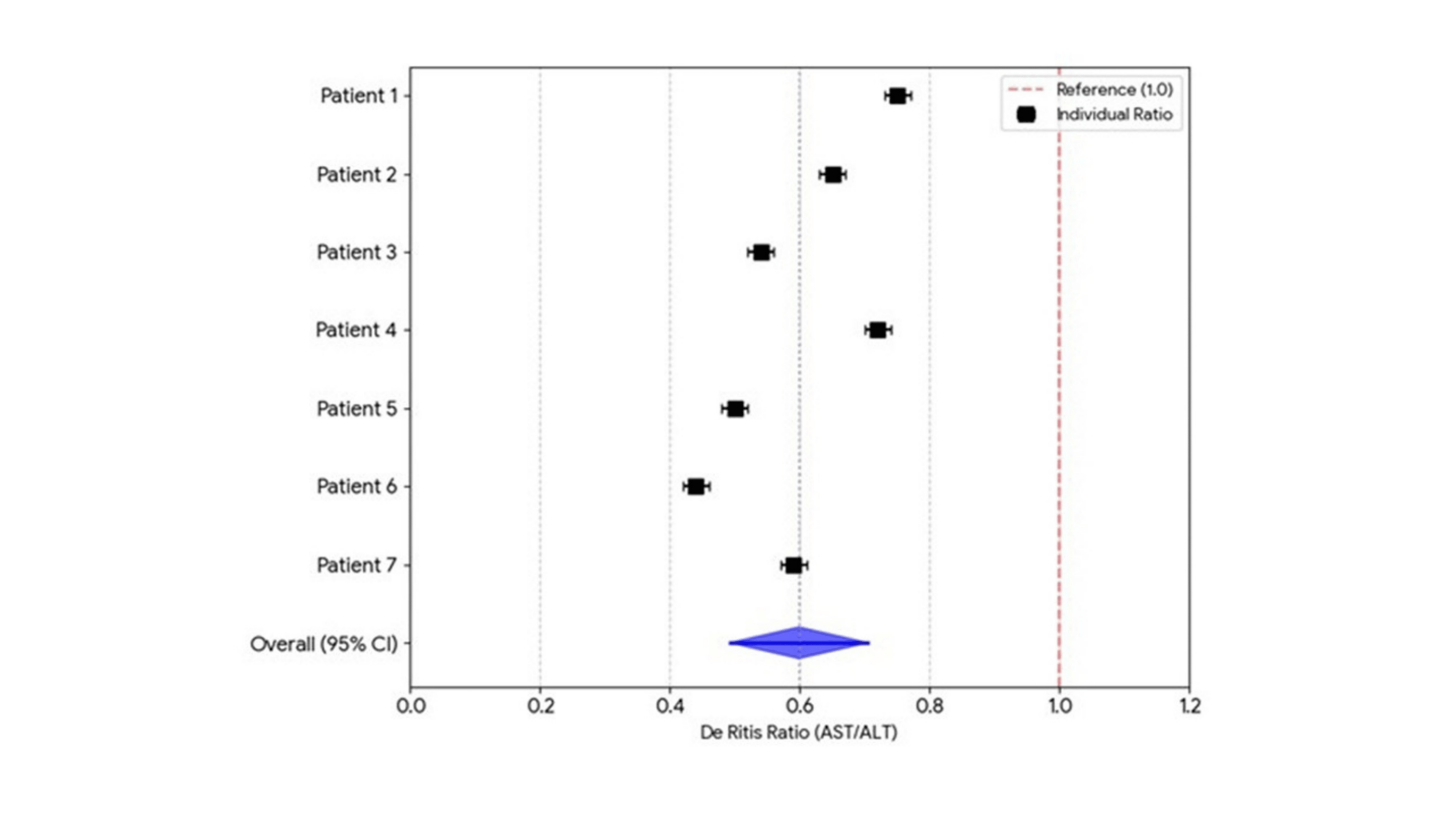
De Ritis ratios in pediatric MASLD cohort. Prior to initiation of therapy each patient exhibited a De Ritis ratio <1. MASLD: metabolically-dysfunction-associated steatotic liver disease; AST: aspartate aminotransferase; ALT: alanine aminotransferase

Prior to initiation of therapy, each patient within this cohort exhibited a de Ritis ratio (AST/ALT) < 1, consistent with a diagnosis of MASLD (Figure 1).

**Figure 2:**
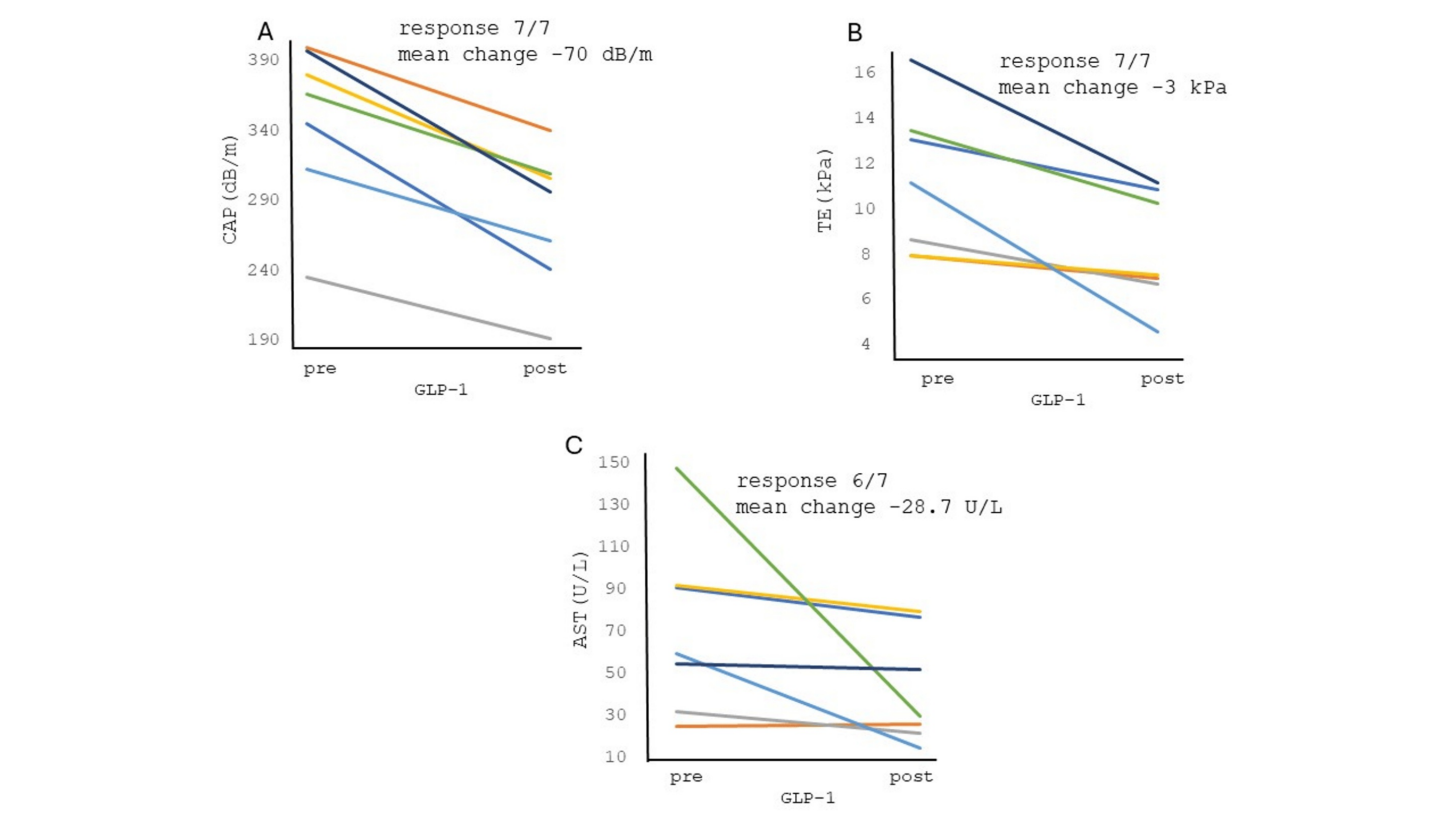
Effects of GLP-1 RAs on liver parameters. Treatment with a GLP-1 RA was associated with a reduction in (A) CAP scores (p=0.0002, paired two-tail T test), (B) TE scores (p=0.01, paired two-tail T test) and (C) AST (p=0.03, non-parametric Wilcoxon Signed Rank Test). Both CAP and TE scores recorded a 100% response rate (p<0.01, Fisher's Exact Test) to therapy whereas 6/7 patients exhibited a reduction in AST levels (p=0.015, Fisher's exact test). GLP-1: glucagon-like peptide-1; RA: receptor agonist; AST: aspartate aminotransferase; ALT: alanine aminotransferase; CAP: controlled attenuation parameter; TE: transient elastography

Administration of a GLP-1 RA was associated with a reduction in liver CAP scores (p=0.0002; Figure 2A), liver TE scores (p=0.01; Figure 2B), and AST levels (p=0.03; Figure 2C). In fact, most patients responded to the drug with a reduction in each of these parameters. The reduction in ALT levels with GLP-1 RA was not significant (p=0.055).

**Figure 3:**
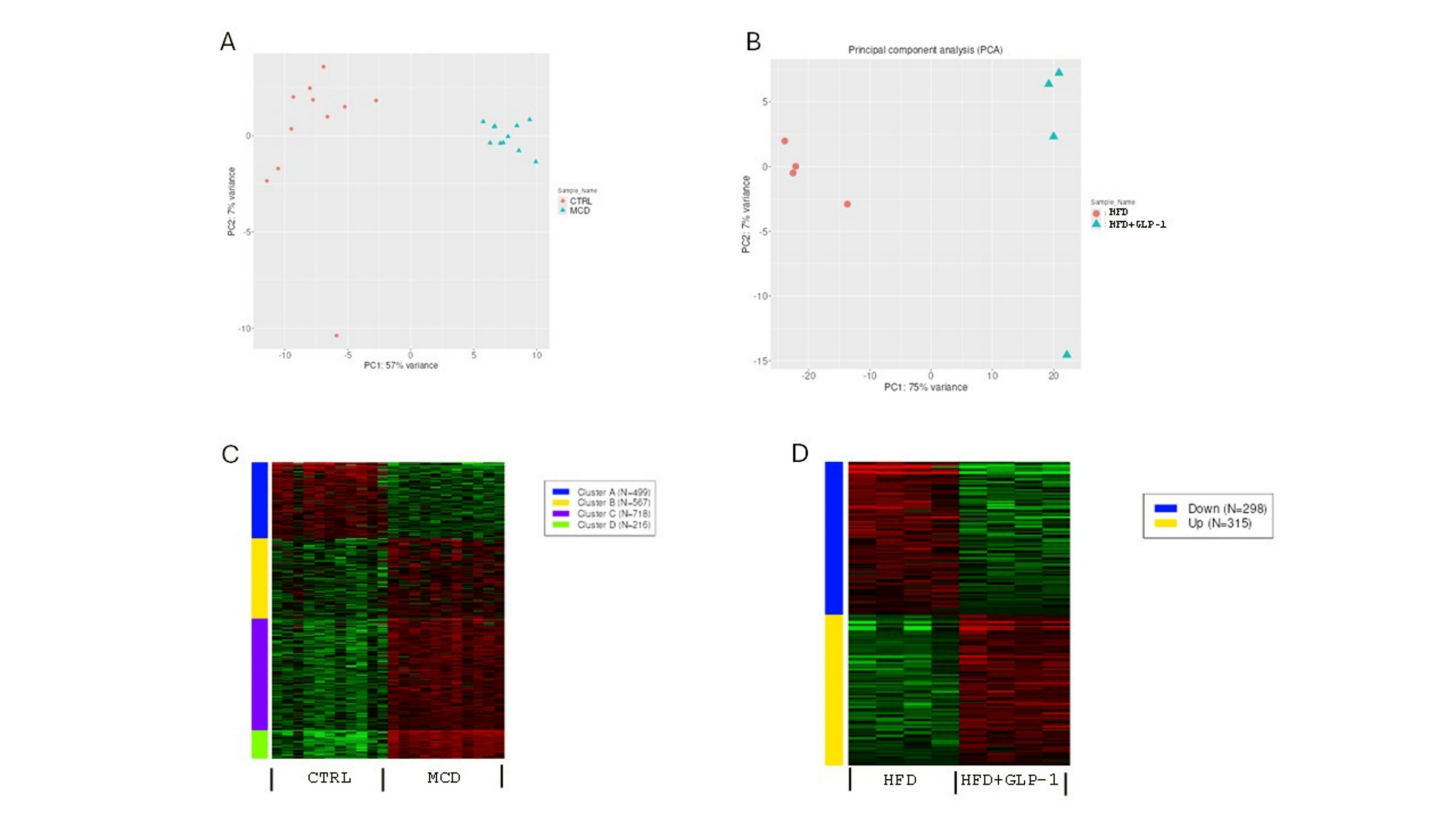
Transcriptomic analysis in murine models of liver disease. PCA plots from MCD (A and B) HFD and HFD+GLP-1 RA models of murine liver disease. (C and D) Up- and down-regulated genes from these models. Study details can be found in GSE267124 and GSE2436. PCA: principal component analysis; MCD: methionine-choline-deficient; HFD: high-fat diet; GLP-1: glucagon-like peptide-1; RA: receptor agonist

Liver TE scores are a surrogate for liver scarring [21], and treatment with GLP-1 RAs was associated with a reduction in this score. We sought to delineate the mechanism of action of GLP-1 RAs using murine models of liver disease. Analysis of datasets GSE267124 (MCD diet model) and GSE243681 (HFD and HFD+GLP-1 RA model) revealed a distinct separation between treatment groups (control vs. MCD diet, Figure 3A; HFD vs. HFD+GLP-1 RA, Figure 3B) and both up- and downregulated genes (Figures 3C, 3D).

**Figure 4:**
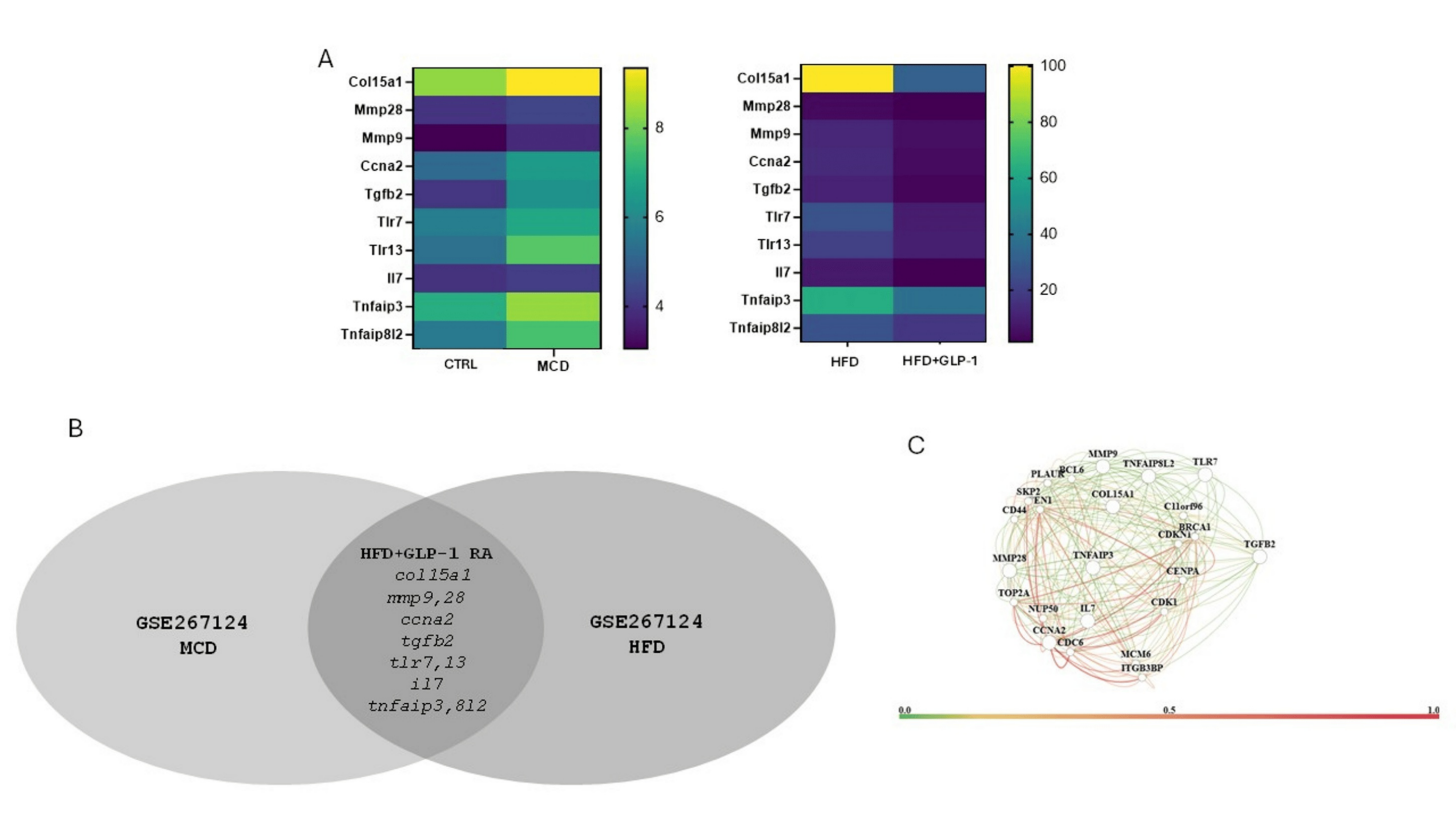
Core gene identification in MASLD models. (A and B) A set of 10 genes was upregulated in the MCD diet and HFD models with expression levels reversed with GLP-1 RA treatment in the HFD model. (C) Seeding these genes into HumanBase revealed a robust interactome in liver tissue. MASLD: metabolically-dysfunction-associated steatotic liver disease; MCD: methionine-choline-deficient; HFD: high-fat diet

A pattern-matching algorithm identified 10 core genes significantly upregulated in both models of liver disease and downregulated with GLP-1 RA treatment in the HFD model (Figures 4A, 4B). Seeding these genes into HumanBase indicates that they form a robust interactome in the liver (Figure 4C).

Additional evidence for the role of these genes in MASLD was derived by seeding them into GepLiver. Expression levels of 9 out of 10 of these genes are elevated in models of murine MASLD (Table 2).

**Table 2:** Core genes identified in our analysis are overexpressed in other murine models of MASLD.

Gene	Healthy mice liver mRNA expression nomalized	MASLD mice liver mRNA expression nomalized
tgfß2	0.29	1.17
col15a1	4.08	5.71
mmp9	0.34	1.04
mmp28	0.13	0.42
il7	0.17	0.44
tlr7	1.28	6
ccna2	0.83	2.31
tnfaip3	1.47	7.74
tnfaip8l2	3.04	6.53

To determine the biological pathways associated with GLP-1 RA activity in MASLD, the core genes were seeded into GOBP. Treatment with GLP-1 RA was associated with restitution of extracellular matrix (ECM) remodeling, matrix deposition, profibrotic and proinflammatory signaling, and a dampening of a heightened injury response (Table 3).

**Table 3:** Mechanisms underlying the activity of GLP-1 RA in a MASLD model. Up arrows represent genes whose levels were upregulated with disease in the MCD and HFD models. Down  arrows represent genes whose levels were downregulated with GLP-1 RA treatment in the HFD model. GLP-1: glucagon-like peptide-1; RA: receptor agonist; MCD: methionine-choline-deficient; HFD: high-fat diet; MASLD: metabolically-dysfunction-associated steatotic liver disease

Gene	Pathway	MCD model	HFD model	HFD+GLP-1 model
col15a1, mmp9,28	ECM remodeling, active basement membrane disruption, collagen deposition	⬆️	⬆️	⬇️
tgfß2	Profibrotic signaling	⬆️	⬆️	⬇️
tlr7,13, il7	Innate immune sensors, cytokine signaling, inflammatory environment	⬆️	⬆️	⬇️
ccna2, tnfaip3,8l2	Comensatory response to injury	⬆️	⬆️	⬇️

## Discussion

This study integrates clinical observations from a small pediatric MASLD cohort with transcriptomic analyses from experimental models of liver disease to explore the therapeutic potential and potential mechanistic basis of GLP-1 RA therapy. Our findings demonstrate that treatment with GLP-1 RAs is associated with significant reductions in both hepatic steatosis (CAP score) and liver stiffness (TE score), suggesting improvements in both metabolic and fibrotic components of MASLD. Importantly, all patients in the cohort exhibited improvement in elastography-based measures, indicating a consistent clinical response despite the small sample size. To the best of our knowledge, this is the first report documenting a reduction in liver stiffness with a GLP-1 RA in a pediatric MASLD cohort.

A validated non-invasive surrogate for hepatic fibrosis, TE scores are increasingly used in both clinical and research settings to monitor disease progression and treatment responses in MASLD and related conditions [25]. Reductions in TE scores following GLP-1 RA therapy therefore suggest that these agents may not only reduce hepatic fat accumulation but also mitigate early fibrotic remodeling within the liver. Similar improvements in hepatic steatosis and fibrosis markers have been reported in adult MASLD and cohorts treated with GLP-1-RAs, supporting the translational relevance of our observations [26].

To explore the molecular basis for these clinical effects, we analyzed transcriptomic datasets from murine models of diet-induced liver disease. A cross-model pattern-matching strategy identified a core group of 10 genes that was consistently upregulated during liver injury and suppressed following GLP-1 RA treatment. Indeed, use of the GepLiver search engine [21] supported the roles of these core genes across murine MASLD models. The 10 core genes identified in this analysis*, tgfβ2*, *col15a1*, *mmp9*, *mmp28*, *il7*, *tlr7*, *ccna2*, *tnfaip3*, *tnfaip8l2*, and *tlr13, represent* a coordinated transcriptional program associated with liver injury, inflammation, and extracellular matrix remodeling [27]. Many of these genes are linked to hepatic stellate cell activation, fibrogenic signaling, and immune pathway activation, which are central drivers of MASLD progression and fibrosis development [27]. The fact that expression of this gene set was consistently elevated across multiple liver disease models and reversed following GLP-1 receptor agonist treatment suggests that this core gene network may represent a conserved molecular signature of liver injury that is responsive to metabolic therapy. These data engender the hypothesis that these genes may serve as mechanistic indicators of GLP-1 activity in human MASLD, whilst also being potential biomarkers for disease activity or therapeutic response in human MASLD. Network analysis demonstrated that these genes form a tightly connected interactome in liver tissue, suggesting that they participate in coordinated regulatory pathways associated with disease progression. Importantly, pathway enrichment analyses revealed that these genes are strongly associated with ECM remodeling, collagen deposition, inflammatory signaling, and injury response pathways-all hallmarks of HSC activation and fibrogenesis [27].

Activation of HSCs represents a central event in the development of liver fibrosis [27]. Upon liver injury, stellate cells transition from a quiescent state to a myofibroblast-like phenotype characterized by increased production of extracellular matrix proteins such as collagen and fibronectin [27]. Persistent activation of this program leads to excessive matrix deposition, tissue stiffening, and architectural distortion of the liver. The suppression of genes associated with ECM production and inflammatory signaling observed in the GLP-1 RA-treated model provides a plausible mechanistic explanation for the reductions in liver stiffness observed in the clinical cohort.

Several biological mechanisms may contribute to these transcriptional effects. GLP-1 RA improves insulin sensitivity, reduces hepatic lipotoxicity, and promotes weight loss, all of which reduce metabolic stress within hepatocytes [28]. In addition, GLP-1 signaling has been shown to suppress inflammatory cytokine production and oxidative stress, both of which are key drivers of stellate cell activation [29]. Furthermore, GLP-1-based therapies may exert direct hepatic effects through modulation of lipid metabolism, mitochondrial function, and immune cell signaling within the liver microenvironment [30].

Limitations of this study should be acknowledged. The primary limitation of the present study is the small clinical cohort size, conducted in a single center, which limits the generalizability of the results. Although the observed responses were statistically significant and consistent across patients, larger multi-center randomized, placebo-controlled studies will be required to confirm these findings. Second, the use of the type of GLP-1 RA was not uniform across all patients, with one subject receiving GLP-1 RA+GIP treatment. Finally, the mechanistic analyses were derived from murine transcriptomic datasets rather than human liver samples and are therefore at best hypothesis-engendering in terms of their extension to human data. Future studies should evaluate gene expression changes directly in pediatric MASLD patients receiving GLP-1 RAs to determine whether the transcriptional signatures observed in murine models are conserved in humans. Longitudinal biopsy or circulating transcriptomic studies may provide further insight into the molecular processes underlying the therapeutic response. Nevertheless, the integration of clinical outcomes with system-level transcriptomic analyses provides a framework for identifying molecular pathways associated with this response. By linking improvements in liver stiffness to the suppression of fibrogenic gene networks, this study highlights potential transcriptional programs that may serve as biomarkers or therapeutic targets in MASLD.

## Conclusions

This study for the first time provides evidence that GLP-1 RA therapy is associated with improvements in hepatic steatosis and liver stiffness profiles in pediatric patients with MASLD. Integrative transcriptomic analyses from murine models of liver disease identified a core gene signature linked to inflammatory signaling that is reversed by GLP-1 RA treatment. These findings suggest that GLP-1 RAs may exert therapeutic effects not only through metabolic improvements and engender the hypothesis that they attenuate transcriptional programs that drive human hepatic fibrosis. Adapting aspects of the four-pillar strategy in place for treatment of type 2 diabetes and chronic kidney disease and adult MASLD may represent an effective therapeutic modality for the treatment of pediatric MASLD.
